# High-fat diet-negative impact on female fertility: from mechanisms to protective actions of antioxidant matrices

**DOI:** 10.3389/fnut.2024.1415455

**Published:** 2024-06-10

**Authors:** Chiara Di Berardino, Urte Barceviciute, Chiara Camerano Spelta Rapini, Alessia Peserico, Giulia Capacchietti, Nicola Bernabò, Valentina Russo, Valentina Gatta, Fani Konstantinidou, Marisa Donato, Barbara Barboni

**Affiliations:** ^1^Department of Bioscience and Technology for Food, Agriculture and Environment, University of Teramo, Teramo, Italy; ^2^Institute of Biochemistry and Cell Biology (IBBC), National Research Council, Rome, Italy; ^3^Department of Psychological Health and Territorial Sciences, School of Medicine and Health Sciences, “G. d'Annunzio” University of Chieti-Pescara, Chieti, Italy; ^4^Unit of Molecular Genetics, Center for Advanced Studies and Technology (CAST), “G. d'Annunzio” University of Chieti-Pescara, Chieti, Italy

**Keywords:** high-fat diet, biological matrix, antioxidant, ovarian health, folliculogenesis, oocyte quality, embryo development

## Abstract

**Introduction:**

Excessive calorie intake poses a significant threat to female fertility, leading to hormonal imbalances and reproductive challenges. Overconsumption of unhealthy fats exacerbates ovarian dysfunction, with an overproduction of reactive oxygen species causing oxidative stress, impairing ovarian follicle development and leading to irregular ovulation and premature ovarian failure. Interest in biological matrices with high antioxidant properties to combat diet-related oxidative stress has grown, as they contain various bioactive factors crucial for neutralizing free radicals potentially preventing female reproductive health. This systematic review evaluates the female reproductive impact of biological matrices in mitigating oxidative damages induced by over calory habits and, in particular, high fat diets.

**Methods:**

A comparative approach among mammalian models was utilized to interpret literature available data. This approach specifically investigates the antioxidant mechanisms of biological matrices on early and late ovarian folliculogenesis, under physiological and hormone-induced female reproductive cycle. Adhering to the PRISMA 2020 guidelines, only English-language publications from peer-reviewed international indexes were considered.

**Results:**

The analysis of 121 publications meeting the inclusion criteria facilitated the identification of crucial components of biological matrices. These components, including carbocyclic sugars, phytonutrients, organosulfur compounds, and vitamins, were evaluated for their impact on ovarian follicle resilience, oocyte quality, and reproductive lifespan. The detrimental effects of oxidative stress on female fertility, particularly exacerbated by high saturated fat diets, are well-documented. *In vivo* studies across mammalian preclinical models have underscored the potential of antioxidants derived from biological matrices to mitigate diet-induced conditions. These antioxidants enhance steroidogenesis and ovarian follicle development, thereby improving oocyte quality. Additionally, discussions within these publications emphasized the clinical significance of these biological matrices, translating research findings into practical applications for female health.

**Conclusion:**

Further research is essential to fully exploit the potential of these matrices in enhancing female reproduction and mitigating the effects of diets rich in fatty acids. This requires intensified *in vitro* studies and comprehensive collection of *in vivo* data before clinical trials. The promotion of ovarian resilience offers promising avenues for enhancing understanding and advancing female reproductive health world-wide.

## 1 Introduction

Dietary choices, with an excessive calorie intake, present substantial threat to female fertility by triggering hormonal imbalances giving rise to reproductive challenges (1–4). Although fatty acids represent essential components of the diet and exert a key role in the body metabolic processes, excessive consumption of unhealthy fats, particularly saturated and trans fats, can potentially contribute to ovarian dysfunction and failure-related pathological conditions (5–8). Going into detail, the dietary-related pathological condition involves an excess production of reactive oxygen species (ROS), resulting in oxidative stress that can harm the synergistic development of somatic and germinal-ovarian follicle compartments, leading to irregular ovulation patterns and premature ovarian failure (9, 10). In this context, diet induced-ROS production can impede vasodilation and blood flow to reproductive organs, disrupting the functioning of the hypothalamic-pituitary-ovarian axis and contributing to (1) impact the reproductive hormones regulation (11–13), (2) induce insulin resistance and hyperleptinemia (14), (3) promote chronic low-grade inflammation (15, 16), (4) affect oocyte quality (13, 17) and (5) influence the maternal uterine environment (18), making it less receptive to embryo implantation and the maintenance of pregnancy (19, 20). To counteract the overall detrimental effects on female reproductive health, the use of antioxidants to mitigate, and potentially reverse, the oxidative stress associated with a high-fat diet could prove to be a beneficial strategy (13, 18, 19, 21–25).

In recent years, research on biological matrices with high antioxidant properties, has garnered significant interest due to the growing recognition of the importance of this compound in promoting health and preventing oxidative stress related to an unbalanced diet habit (26–28). More in detail, biological matrices, which can have either plant or animal origins, often contain a variety of antioxidant compounds such as vitamins, polyphenols, flavonoids, and carotenoids, playing a crucial role in mitigating the oxidative damage caused by free radicals in the body. Literature studies have demonstrated that a diet rich in foods containing these biological matrices can help reduce the risk of oxidative-related conditions, supporting healthy ovarian follicle growth and overall reproductive health (29).

Considering that the interest in these biological matrices stems from the growing awareness of the importance of a diet rich in antioxidants for promoting female reproductive wellbeing, the aim of this systematic review is to assess the impact of biological matrices on controlling oxidative exposure due to a high caloric food intake and, in turn, its potential influence on ovarian function. The employment of a comparative approach amongst mammalian models during folliculogenesis has been proposed for interpreting the vast quantity of data gathered so far. Specifically, it focuses on the study of antioxidant mechanisms of action of biological matrices and their beneficial properties in relation to the physiological processes associated with early and late ovarian folliculogenesis, either under physiological or hormone-induced conditions.

## 2 Methods

### 2.1 Bibliographic search methods

The present systematic review was carried out following the Preferred Reporting Items for Systematic Review and meta-analysis (PRISMA) Statement 2020 Checklist Guidelines (http://www.prisma-statement.org/) (30).

Scientific literature published in the peer-reviewed international index was considered. Specifically:

- Advanced Search of Web of Science [v.5.35] “Core collection” archive (https://apps.webofknowledge.com/WOS_AdvancedSearch).
- PubMed Advanced Search Builder archive (https://pubmed.ncbi.nlm.nih.gov/advanced/) was considered.

“TS” was used as a Field tag, “AND,” “OR,” and “NOT” were used as Boolean operators.

The keywords were combined to elaborate the main paragraphs, as follow:

- List: “WOS Advanced Search”

((((TS = (oxidative stress)) AND TS = (biological antioxidant^*^)) AND TS = (female reproduction)) AND TS = (mammal^*^)) NOT TS = (male)

(TS = (oxidative stress)) AND TS = (ovarian failure)

(TS = (antioxidant^*^)) AND TS = (ovarian folliculogenesis)

((TS = (high fat diet)) AND TS = (antioxidant)) AND TS = (ovary)

(TS = (biological antioxidant)) AND TS = (ovary)

(TS = (biological matrix)) AND TS = (ovarian damage)

(TS = (biological matrix^*^)) AND TS = (ovarian folliculogenesis)

(TS = (diet)) AND TS = (female infertility)

Matched list: 925 publications.

- List: “PubMed Advanced Search”

((((oxidative stress) AND (high fat diet)) AND (female reproduction)) AND (mammal^*^)) NOT (male)

(folliculogenesis) AND (high fat diet)

((fertility) AND (high fat diet)) AND (antioxidant^*^) NOT (male)

(((ovarian follicle^*^) AND (high fat diet)) AND (antioxidant^*^)) NOT (male)

(((high fat diet) AND (antioxidant)) AND (follicle^*^)) NOT (male)

((high fat diet) AND (oxidative stress)) AND (ovarian follicle^*^)

(((high fat diet) AND (follicle^*^)) AND (oxidative stress)) NOT (male)

(antioxidant^*^) AND (folliculogenesis) AND (fat)

(antioxidant^*^) AND (folliculogenesis) AND (obese) AND (mammal)

(antioxidant^*^) AND (ovarian follicle) AND (obese) AND (mammal) NOT (male)

Matched list: 80 publications.

### 2.2 Eligibility criteria

Only English-language publications were considered. The reviewed articles, which investigated the connection between high-fat diets, ROS production, and female fertility in relation to follicle development, steroid production, and oogenesis, were published from 1971 to 2024. The initial screening of studies was primarily based on their titles and accompanying abstracts. Except for articles focused on obesity, research papers that did not explicitly address the topics of “high-fat diet” and “oxidative stress” related to fertility were excluded. Studies involving non-mammalian species or male animal models were ultimately removed from consideration.

### 2.3 Study selection

Considering the information provided above, each list was subsequently cross-referenced, and the initial count of titles, in accordance with the search keywords, was estimated to be 1,005. Duplicate were eliminated. Comprehensive manuscripts were then obtained for all the chosen documents, and the final selection was made after a thorough evaluation.

## 3 Results

**Figure 1:**
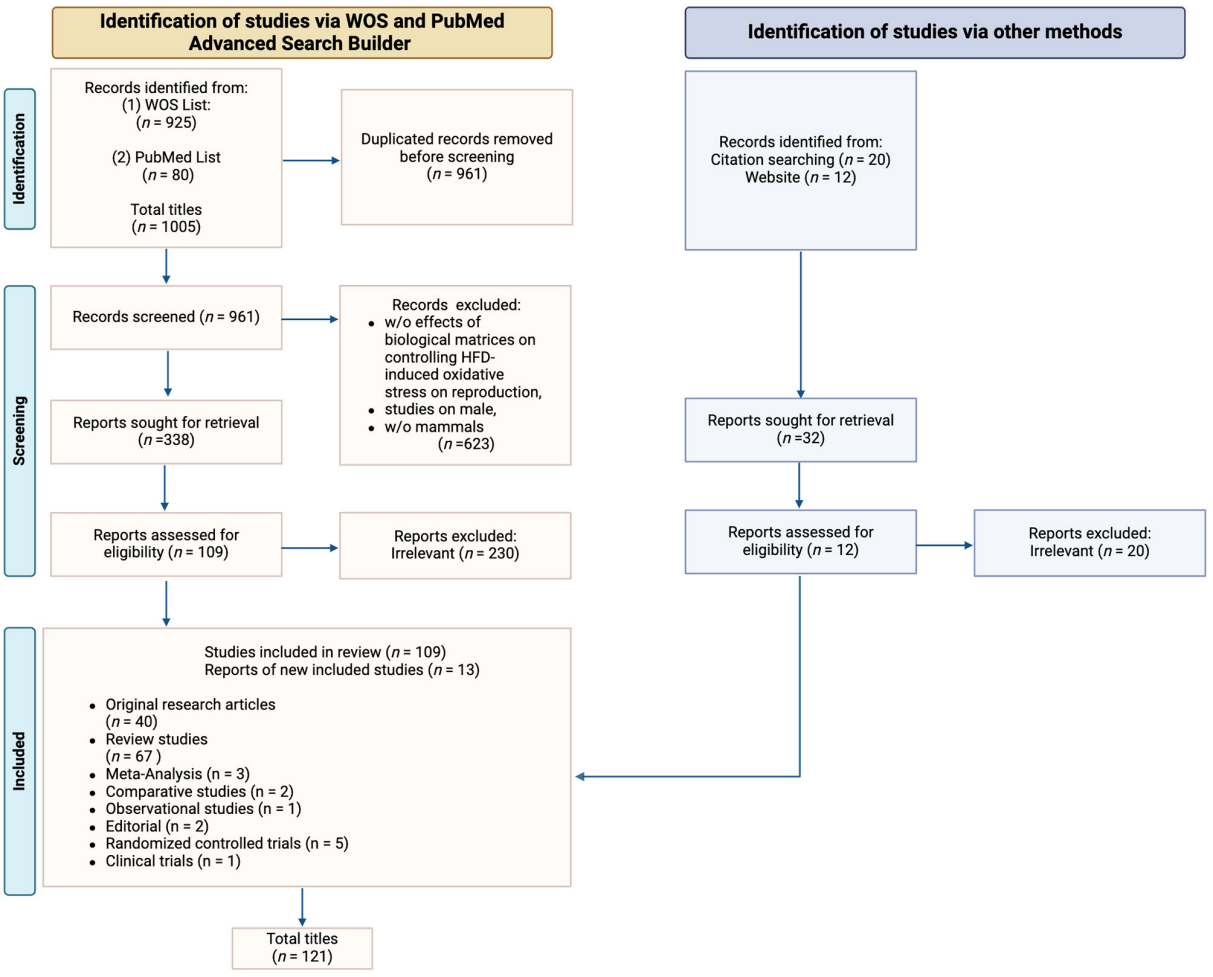
PRISMA flow diagram. The diagram shows the systematic process adopted to include papers captured by the literature search. Preferred Reporting Items for Systematic Review and meta-analysis” (PRISMA) Statement 2020 Checklist Guidelines were followed. Image created with Biorender.com.

Original research articles (*n* = 40), meta-analysis (*n* = 3), comparative studies (*n* = 2), observational studies (*n* = 1), editorial (*n* = 2), randomized controlled trials (*n* = 5) and clinical trials (*n* = 1) were chosen to investigate the impact of diet induced-ROS production on specific mammalian models and to evaluate their reproductive outcomes. Review articles (*n* = 67) were included solely for the purpose of enhancing and discussing the gathered data. Their reference lists were also examined and analyzed, alongside a comprehensive website check, to identify other studies or potentially related information that could be further included in this review. Ultimately, 121 publications conformed to the inclusion criteria (Figure 1). As a note, in the systematic selection of the aforementioned articles, the reference related to the proper utilization of the PRISMA Statement 2020 Checklist Guidelines (30) is excluded from the count.

### 3.1 Biological matrices-derived antioxidants as dietary components: their impact on ovarian health and their role in modulating the intricate relationship between high-fat diet-induced oxidative stress and ovarian function

The intricate relationship between high-fat diet (HFD)-induced oxidative stress and ovarian function has profound implications for the ovarian microenvironment (4, 31). The ovarian microenvironment is a complex and dynamic milieu within the ovaries that plays a crucial role in supporting normal ovarian function and reproductive health (32). It encompasses the cellular, biochemical, and physical factors that influence the growth and health of ovarian follicles and their enclosed oocytes (33). Maintaining the integrity of the ovarian microenvironment is essential for a proper hormonal regulation (34), follicular development (35) and oocyte maturation (36), mitochondrial function within the oocytes (37), vascularization and blood supply (38–41), immunological regulation (42), as well as fertility and reproductive potential (33, 43, 44). However, the disruptions caused by oxidative stress can have far-reaching consequences on the ovarian microenvironment, impacting the overall mechanisms regulating ovarian function and female reproductive health (45, 46). More in detail, in a healthy organism, ROS and antioxidants remain in balance. When the balance is disrupted toward an overabundance of ROS due to a high levels of fat intake, oxidative stress occurs, determining metabolic dysfunctions that can impair the entire reproductive span of female's life (45, 47). Cells have developed a wide range of antioxidants systems to limit production of ROS, inactivate them and repair cell damage (48–50). These systems are based on the use of enzymatic and non-enzymatic antioxidant systems to protect against ROS and subsequent damage to membranes and macromolecules. Despite the presence of endogenous enzymatic antioxidants, composed of superoxide dismutase, catalase, glutathione peroxidase and glutathione reductase, that catalyze reactions to neutralize free radicals (4, 5), nowadays the role and the biological action of biological matrices as exogenous antioxidants are under investigation, considering the potential role of these dietary supplements in help reverse or mitigate the effects of HFD induced-oxidative stress on ovarian health (51–53).

More in detail, biological matrices that have a positive influence on female reproductive health include carbocyclic sugar (54), phytonutrients (55–57), organosulfur compound (54, 58), hormones (59, 60), neuropeptide (61), organic acids (62), and vitamins (58). On the contrary, components exerting a negative influence mostly include high glycemic index carbohydrates, large amount of animal protein, saturated fatty acids, and trans fatty acids, which are typical of the Western model of nutrition, which is closely related to increased oxidative stress (4, 5, 53).

The research on the role of diet and other modifiable factors associated with lifestyle in the prophylaxis of fertility disorders is still ongoing. However, few studies have established a direct link between nutrition and the risk of anovulatory infertility. Therefore, the aim of the following sections was to summarize available knowledge about diet and its relation to anovulatory infertility and indicate avenues for future research. Considering this, the following paragraphs aim to provide a comprehensive analysis across mammal species of the impact that a balanced diet supplemented with antioxidants derived from biological matrices can have on female reproductive health. These sections also endeavor to gather crucial information from the literature regarding the mechanisms by which biological matrices counteract the detrimental effects of a diet rich in fatty acids on female reproductive wellbeing, with a particular emphasis on ovarian follicle development, oocyte quality, and reproductive lifespan.

A comprehensive overview delineating the effects of a balanced diet enriched with biologically derived antioxidants to mitigate the adverse impact of HFD on female reproductive health across mammal species was depicted in Supplementary Figure 1.

#### 3.1.1 Biological matrices-derived antioxidants in HFD-induced oxidative stress: implications for ovarian follicle development

Folliculogenesis allows primordial germ cells mature within follicles into oocytes capable of fertilization and embryonic development, which is crucial for reproductive success (63). Ovarian follicle development, which is critical part of female reproductive health, is increasingly recognized as susceptible to dietary influences, one of which is causing oxidative stress (4). In fact, HFD has been found to result in an excessive availability of energetic precursors, such as glucose, pyruvate, and other metabolic intermediates. Additionally, it leads to the accumulation of intracellular lipids that bind to proteins, forming lipid droplets, thus causing lipotoxicity (4, 63). These precursors are metabolized through the glycolytic pathway and the respiratory chain to produce energy in the form of ATP, which in turn causes impairment in ovarian follicle development (64). The accumulation of intracellular lipids leads to high levels of free fatty acids, which are susceptible to oxidative damage and the formation of cytotoxic and highly reactive lipid peroxides. Ultimately, these compounds are detrimental to intracellular organelles, particularly the endoplasmic reticulum (ER) and mitochondria (3, 5). Thus, HFD can cause oxidative stress on the ovary, specifically targeting follicle development, survival, and hormone production essential for regulating folliculogenesis, which in turn negatively impacts female fertility (5).

There are several pathways by which oxidative stress inflicts damage, such as reactive oxygen species (ROS) formation, diminished antioxidant capacity in the ovary, disrupted mitochondrial function and several others reported in recent literature outlined in Table 1 with further details available in Supplementary Table 1. The oxidative stress profoundly impacts not only follicle formation, growth, and maturation, but also oocyte quality, potentially compromising embryo development (64). The results described in Table 1 demonstrate that while under normal physiological and hormonal conditions follicles progress to yield healthy oocytes, oxidative stress induced by HFD can disrupt this process at any point, impairing steroidogenesis in addition to affecting folliculogenesis as well as oocyte quality, and therefore reproductive lifespan.

**Table 1:** An overview of the current literature survey on antioxidant treatments for HFD-induced oxidative stress on ovarian function in mammalian models.

**Lipid diet composition**	**Dietary supplements**			**Mechanism of action and biological effect**	**References**
	**Supplement**	**Bioactive compound category**	**Dose of supplement**		
HFD (*diet composition N/D*)	Myo-inositolα-lipoic acid	Carbocyclic sugarOrganosulfur compound	800 mg α-lipoic acid, 2 g myo-inositol/day	Decreased levels of ROS and enhanced total antioxidant capacity in follicular fluid, leading to an improved oocyte environment	(54)
Standard diet + 3% cholesterol, 8% cocoa butter, 2% cholic acid, 1% thiouracil and 1% starch	Barley (*Hordeum vulgare*) and dates (*Phoenix dactylifera*)	Phytonutrients	Either 10% barley grains, 10% date palm fruit, or a combination of both (10% dates and 10% barley)	Enhanced proliferation and maturation of healthy ovarian follicles, restoration of nearly normal cellular and structural patterns in the ovarian stroma, mitigation of oxidative stress and lipid peroxidation within the ovary, refinement, and amelioration of antioxidant levels in the assayed enzymes and sex steroid hormones	(55)
HFD (*diet composition N/D*)	Gum Arabic (*Acacia senegal*)	Phytonutrients (fiber)	10% weight/weight of gum arabic (GA)	The intervention led to heightened activities of antioxidant enzymes such as superoxide dismutase, catalase, and glutathione peroxidase in the ovaries. Consequently, it mitigated oxidative stress and lipid peroxidation. Additionally, there was an increase in mRNA expression of antioxidant enzymes within the ovaries, which contributed to safeguarding against degenerative changes	(65)
HFD (*diet composition N/D*)	Resveratrol	Phytonutrients (phenol)	10 mg/kg/day for 3 weeks	Enhanced oocyte meiosis, reinstated spindle assembly, and mitigated oxidative stress and irregular mitochondrial distributions within the oocyte. Furthermore, it restored the mechanical properties of oocytes, specifically the hardness of the zona pellucida	(56)
The fat content of HFD was adjusted to 60% by addition of beef tallow into standard diet	Okra (*Abelmoschus esculentus*)	Phytonutrients (flavonoid)	200 mg/kg for 30 days	The intervention led to a decrease in the numbers of atretic preantral and antral follicles in the ovary, accompanied by an increase in the activities of antioxidant enzymes such as glutathione peroxidase (GPX) and catalase (CAT). Furthermore, there was a reduction in lipid peroxidation levels and modulation of apoptotic gene expression in the ovaries	(57)
HFD comprising 40% fat, 20% protein, 36% carbohydrate, and 4% others (530 kcal)	Ferulic acid, kaempferol, malvidin, caffeoylquinic acid, and quercetin derivatives extracted from bitter cumin (*Centratherum anthelminticum*) using an ethanolic extraction method	Phytonutrients (flavonoids and phenolic compounds)	250, 500, or 750 mg/kg/day for 28 days	The intervention resulted in improved oxidative stress markers and catalase activity in the ovary, normalized estrous cycle, and balanced reproductive hormone levels	(66)
HFD (*diet composition N/D*)	Thymoquinone	Phytonutrients	TQ (10% pmm) and TQ (20% pmm)	The activated genes associated with the AMPK/PGC1α/SIRT1 pathway exerted a positive influence on oxidative status, leading to a reduction in inflammatory markers and enhancement of mitochondrial function in ovarian tissue. Additionally, they regulated oxidative stress biomarkers and antioxidant enzyme activities	(67)
HFD containing 60% kcal energy prepared using lard	Apple vinegar	Organic acid	5 g vinegar powder/100 g for 8 weeks	The intervention led to an increase in the numbers of primordial and primary follicles in the ovary, as well as elevated serum levels of estradiol. There was a four-fold increase in serum total antioxidant capacity (TAC), and regulation of kisspeptin expression in the ovary was observed, along with its indirect effects on folliculogenesis	(62)
HFD, provided 5.4 kcal/g and consisted of 25.9% carbohydrates, 14.9% proteins, and 59.0% fat	Phoenixin	Peptide (neuropeptide)	Administering 100 nmol/g body weight via gastrogavage for a duration of 10 weeks	The intervention improved obesity-induced infertility by modulating mitochondrial dynamics, resulting in decreased serum levels of insulin and testosterone, as well as ovarian levels of certain proteins and markers associated with oxidative stress and apoptosis. Additionally, there was an increase in serum estrogen, progesterone, luteinizing hormone (LH), and follicle-stimulating hormone (FSH), along with elevated ovarian levels of GnRH receptor (GnRHR), mitofusin2 (Mfn2), mitochondrial transmembrane potential (ΔΨm), and electron transport chain (ETC) complex-I	(61)_
High-fat/sucrose Western diet (HF; TD.190341) has a composition of 15.3% kcal from protein, 42.8% kcal from carbohydrates (with sucrose contributing 345 g/kg), and 41.9% kcal from fat. This diet provides 4.56 kcal/g	α-lipoic acid	Organosulfur compound	1 g/kg for 6 weeks	The improved reproductive success in obesity may be attributed, at least in part, to the mitigation of ovarian inflammation and the reduction of follicular atresia in the ovary	(58)
	Green coffee bean extract	Phytonutrients	2.5 g/kg for 6 weeks		
	Green tea extract	Phytonutrients	0.75 g/kg for 6 weeks		
	Forskolin	Phytonutrients (labdane diterpenoid)	0.125 g/kg for 6 weeks		
	Vitamin E/α tocopheryl acetate	Vitamin (Vitamin E)	2.188 g/kg for 6 weeks		
	Beetroot extract	Phytonutrients (betalains)	10 g/kg for 6 weeks		
	CoQ10	Enzyme (ubiquinone)	2.5 g/kg for 6 weeks		
HFD comprised 54.2% standard diet, 16.8% lard, 15% sucrose, 9% casein, 1% minerals, 1% vitamins, and 3% malt dextrin	MitoQ10	Synthetic enzyme	The treatment regimen involved administering 500 μmol/l of MitoQ10 (Sigma-Aldrich; Merck KGaA) daily for 8 consecutive weeks	The intervention reversed endocrine abnormalities, reduced oxidative stress, and improved mitochondrial function. It also led to a decrease in the expression of apoptotic proteins and improvement in reproductive and metabolic features. Additionally, there was a reduction in ROS levels	(68)
HFD (*diet composition N/D*)	Luteolin	Phytonutrients (flavonoid)	The doses administered were 25, 50, and 100 mg/kg intraperitoneally daily	The intervention normalized the estrus cycle, improved ovarian morphology, and balanced serum sexual hormone levels. It demonstrated inhibitory effects on insulin resistance by regulating the PI3K/AKT signaling pathway. Additionally, it restored the activities of antioxidants such as SOD, GPx, CAT, and GSH, and upregulated the Nrf2 pathway, contributing to an enhanced antioxidative response in the ovaries	(69)
HFD (D12492, Research Diets Inc., New Brunswick, NJ)	Melatonin	Hormone (indoleamine)	The treatment regimen involved administering daily oral doses of 30 mg/kg body weight for 3 weeks	The intervention led to reduced reactive oxygen species (ROS) generation and prevented spindle/chromosome anomalies in oocytes. Consequently, it promoted the developmental potential of early embryos. During *in vitro* maturation, it also attenuated oxidative stress and meiotic defects in HFD oocytes through the SIRT3-SOD2-dependent mechanism, which is the site of action of melatonin	(59)
240 g/day soya oil	Melatonin	Hormone (indoleamine)	The intake of 300 g/day of inulin and cellulose until the 19th day of the fourth estrous cycle led to an increase in serum levels of serotonin and melatonin, which were indirectly synthesized after dietary fiber intake	The intervention protected against ovarian follicular atresia, at least partly through gut microbiota-related serotonin-melatonin synthesis. It resulted in decreased numbers of atretic follicles and lowered expression of apoptotic markers in the ovaries	(60)
	Seratonin	Hormone (monoamine neurotransmitter)			
HFD (D12492, Research Diets Inc., New Brunswick, NJ, United States)	Phycocyanin	Phytonutrients (biliprotein from *Spirulina platensis*)	500 mg/kg/day	The intervention ameliorated the level of ovarian antioxidant enzymes, reduced the occurrence of follicular atresia, and improved both the abnormal morphology of the spindle-chromosome complex (SCC) and the abnormal mitochondrial distribution pattern in oocytes. Additionally, it partially reversed obesity-related accumulation of reactive oxygen species (ROS), decreased the number of early apoptotic cells, and normalized the expression of H3K9me3 in oocytes. Furthermore, it prevented ovarian follicular atresia, reduced follicle atresia rates, and improved fertility-related hormone levels	(70)
The Metabolic Syndrome diet was prepared with additional refined palm oil (10%) and coconut oil (15%)	Betalain	Phytonutrients (betalains)	300 mg/kg for 2 months	There is a mild to moderate enhancement in ovarian function characterized by an augmented follicular count and reduced incidence of ovarian cyst formation	(71)
HFD formulated with supplementary refined palm oil (10%), coconut oil (15%), and cholesterol (1%) was prepared	Frankincense (*Boswellia Carterii*)	Phytonutrients (oil extract)	500 mg/kg body weight for 60 days	The detrimental effects of a high-fat diet on reproductive organs were mitigated, evidenced by an increase in follicle count and a reduction in cyst formation	(72)
Isocaloric high-fat/high-sugar (HF/HS) diet was administered using Test Diet 58R3 (TestDiet), comprising 59% fat, 17% sucrose, and 15% protein by weight	CoQ10	Enzyme (ubiquinone)	Administered three times per week, a dosage of 22 mg/kg dissolved in sesame oil was delivered subcutaneously for a duration of 6 weeks	Mitochondrial distribution abnormalities were completely prevented, resulting in an increased percentage of normal spindle and chromosome alignment. Furthermore, there was notable improvement observed in both oocyte mitochondrial distribution and function	(73)_
HFD comprises 40% of calories derived from fat, with a composition of 60% fat, 20% protein, and 20% carbohydrates	Leptin	Protein hormone	N/D	The observations included reduced ovarian weights, diminished peri-ovarian fat pads, modulation of LH receptor positivity, decreased apoptosis and inflammation, along with increased LH receptor positivity in the ovary	(74)

Detailed information is provided in Supplementary Table 1.

N/D, diet composition or effects not defined.

The studies summarized in Table 1 demonstrate encouraging results of antioxidant dietary supplementation in ameliorating HFD-induced damage, ranging from moderately restoring to significantly improving folliculogenesis compared to control groups. The existing literature highlights the potential of antioxidant treatments in relieving HFD-induced oxidative stress, pointing toward future research directions and treatment possibilities that hinge on a more comprehensive understanding of oxidative stress damage and pathways by which it occurs.

Most of the existing studies on this topic predominantly present results from *in vivo* animal models, primarily rodents such as rats or mice, with limited experiments conducted *in vitro*. Moreover, many studies focus on HFD induced oxidative stress or oxidative stress effects on general fertility, but do not specify the combination of these topics to specifically report the effects of HFD induced oxidative stress on female fertility. This limited *in vitro* experimentation poses challenges in definitively attributing improved outcomes to specific treatments as folliculogenesis is a very complex process affected by numerous factors. More distinct and targeted studies in different animal models or even human trials would provide a more comprehensive view of the effects of HFD induced stress on female fertility. Additionally, performing experiments *in vitro* would allow for a more controlled experiment setting, which would in turn lend credibility and integrity to the results.

**Figure 2:**
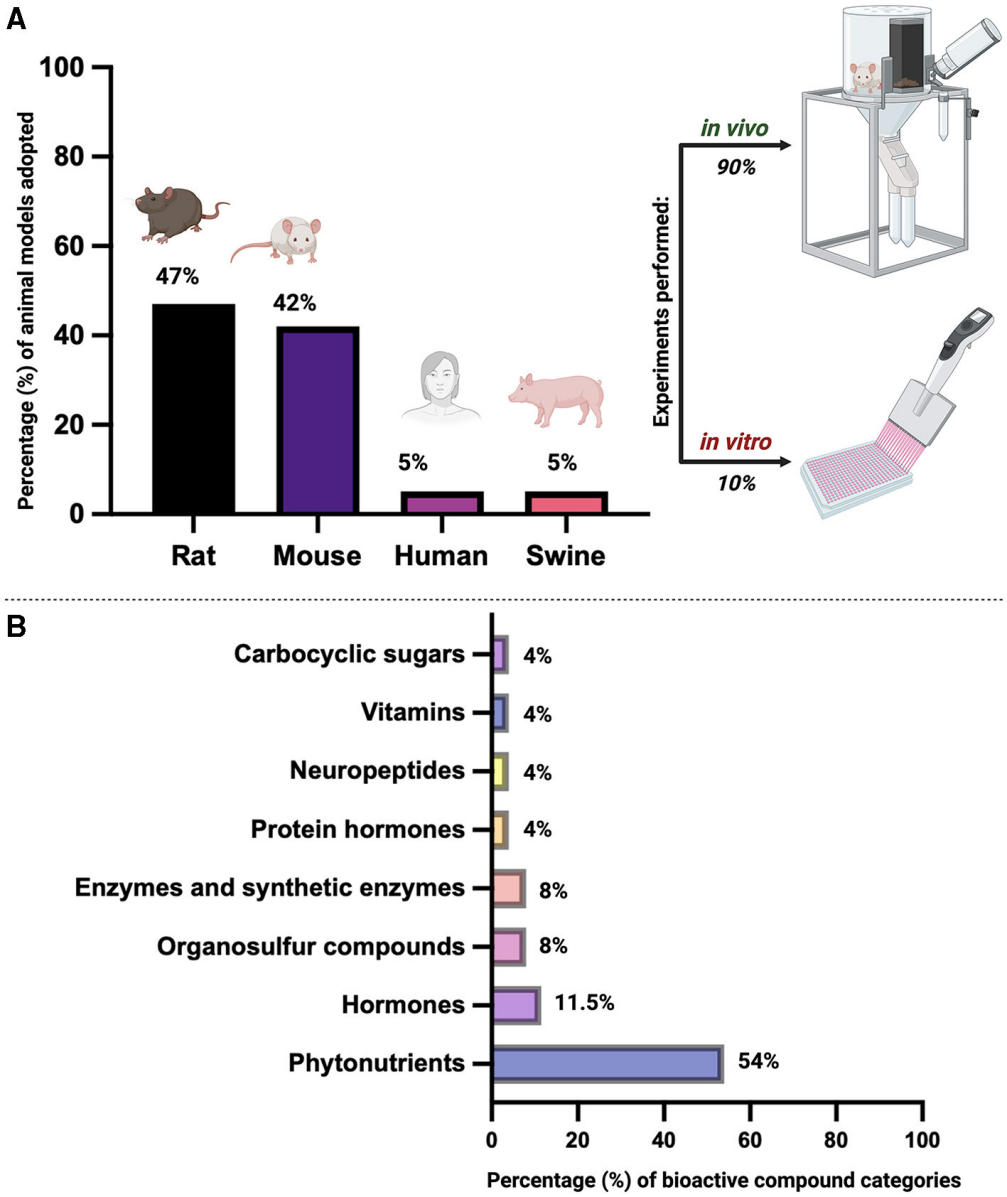
Overview of bibliographic research on the use of biological matrices as antioxidants to counteract the detrimental effect of a High-Fat Diet (HFD) on ovarian folliculogenesis and overall female reproductive health across mammal species. **(A)** Percentage (%) of animal models used for *in vivo* and/or *in vitro* studies. Percentages are calculated based on the literature information reported in Supplementary Table 1. **(B)** Percentage (%) of bioactive compound categories. Percentages are calculated based on the literature information reported in Supplementary Table 1. Image created with Biorender.com.

A detailed summary of the current section was provided in Figure 2.

#### 3.1.2 Effect on early ovarian folliculogenesis

HFD induced oxidative stress causes significant challenges on early ovarian folliculogenesis, especially on follicle growth and survival. However, various studies have elucidated strategies to mitigate these effects using antioxidants as dietary supplements in various animal models which are summarized in Table 1. For example, El-Sayyad et al. observed a significant preservation of ovarian follicles under conditions of oxidative stress induced by a lipid-rich diet. This preservation was accompanied by an increase in the proliferation and development of both primordial and primary follicles, and a restoration of ovarian stroma to near-normal conditions. These effects were achieved through the *in vivo* administration of a diet containing a combination of two phytonutrients—barley (*Hordeum vulgare*) and dates (*Phoenix dactylifera*)—to rodent models (55). Furthermore, the study noted an enhancement in the levels of antioxidant enzymes, effectively mitigating oxidative stress and lipid peroxidation within the ovary. Similarly, other studies, including those by Majd et al. (57) and Shoaib et al. (66), reported enhanced growth of early-stage follicles and a reduction in the rate of atretic follicles. These positive outcomes were attributed to the heightened activities of flavonoids and phenolic-antioxidant properties found in Okra (*Abelmoschus esculentus*) (57), ferulic acid, kaempferol, malvidin, caffeoylquinic acid, and quercetin derivatives (*Centratherum anthelminticum*) (66). These dietary compounds were administered *in vivo* in a rat model, resulting in diminished lipid peroxidation levels.

Moreover, the incorporation of thymoquinone into the diet of mouse models (67) led to the activation of genes associated with the AMPK/PGC1α/SIRT1 pathway. This pathway appears to play a pivotal role in enhancing oxidative status, reducing inflammatory markers in HFD conditions, and boosting mitochondrial function. This ultimately resulted in an increased count of early-stage follicles that were able to reach the stage of Graafian follicles, allowing them to ovulate meiotically competent oocytes (67). Additionally, interventions that target kisspeptin expression via the dietary supplement of apple vinegar in rodent *in vivo* model (62), and those that target serotonin-melatonin synthesis related to gut microbiota in porcine model used as dietary supplements (60), have significantly contributed to the protection against follicular atresia and the promotion of early ovarian folliculogenesis.

Furthermore, numerous studies have pinpointed theca cells and granulosa cells as the primary sites for antioxidant action. For example, leptin treatment in mouse model resulted in a significant expansion of the granulosa and theca cell layers, coupled with increased LHR immunoreactivity (74). This not only accelerated follicular turnover but also boosted the number of early-stage follicles capable of reaching the antral stage.

Concurrently, the incorporation of the neuropeptide phoenixin into the diet of rodents resulted in observable changes, including reduced ovarian weights, decreased peri-ovarian fat pads, and modulated luteinizing hormone (LH) receptor positivity. This dietary intervention also led to a reduction in apoptosis and inflammation within the ovary (61). In a parallel vein, research conducted by Zhuo et al. (60) demonstrated a significant decrease in granulosa cell apoptosis and the proportion of atretic antral follicles. This outcome was partially attributed to the synthesis of serotonin-melatonin related to gut microbiota, which ultimately led to a reduction in atretic follicles and a decrease in the expression of apoptotic markers within the ovaries. Furthermore, the observation of weak positive reactions of caspase-3 and iNOS in the granulosa cells of ovarian follicles and stromal cells suggests an amelioration of the adverse effects of a HFD on reproductive organs, as referenced. This improvement is characterized by an increased count of early-stage follicles and a reduction in the number of cysts following the administration of betalain (71) and frankincense (*Boswellia Carterii*) (72) in a rodent model diet. These comprehensive findings underscore the complex interplay of various pathways and interventions in mitigating the impacts of high-fat-diet-induced oxidative stress on ovarian folliculogenesis, offering promising avenues for future therapeutic strategies.

#### 3.1.3 Effects on late ovarian folliculogenesis under physiological and hormonal-induced conditions

The detrimental impacts of a HFD on late ovarian folliculogenesis under various physiological and hormonal-induced conditions, such as obesity, polycystic ovary syndrome (PCOS), insulinemia, and metabolic syndrome, are substantial. However, research suggests promising avenues for mitigating these effects. For instance, high-fat diet-induced oxidative stress can lead to degenerative changes in the ovary, while antioxidant supplementation in the diet is associated with increased activities of antioxidant enzymes and protection against degenerative alterations, consequently improving PCOS symptoms and restoring reproductive hormone balance (65, 66).

Moreover, Nilsson et al. employed a multi-antioxidant supplement, including organosulfur compound (α-lipoic acid), phytonutrients (forskolin, extracts of green coffee bean, green tea, and beetroot), vitamin E (α-tocopheryl acetate), and Coenzyme Q10 (CoQ10), in an obese rat model. Their study demonstrated promising results in mitigating obesity-induced infertility by reducing ovarian inflammation and follicular atresia, consequently enhancing reproductive success (58). Moreover, targeted treatments such as MitoQ10 supplementation (68) and melatonin supplementation acting via the SIRT3–SOD2-dependent mechanism (59) demonstrate the potential to reverse endocrine abnormalities, reduce oxidative stress, and improve mitochondrial function, consequently ameliorating obesity-induced reproductive defects and promoting oocyte developmental potential. Additionally, modulation of hormonal levels, including FSH, LH, estrogen, and progesterone, plays a crucial role in restoring ovarian function. Studies conducted by Basha et al. (61) and Shams et al. (62) suggests that the hormonal imbalances caused by a HFD can be rectified in rat models through the administration of a diet containing apple vinegar and phoenixin. This dietary intervention appears to enhance folliculogenesis and bolster antioxidant responses within the ovaries. Collectively, these findings show the intricate interplay between high-fat diet-induced oxidative stress, hormonal imbalances, and ovarian folliculogenesis, while offering insights into potential therapeutic interventions to mitigate these adverse effects and restore reproductive health.

#### 3.1.4 Biological matrices-derived antioxidants in HFD-induced oxidative stress: implications for oocyte quality

The harmful effects of a high-fat diet on oocyte quality, particularly concerning spindle formation, chromosome alignment, meiotic defects, mitochondrial function, and DNA damage, pose significant challenges to reproductive health. Studies elucidate the intricate interplay between high-fat diet-induced oxidative stress and various aspects of oocyte quality. For instance, HFD-induced oxidative stress can impair oocyte spindle formation and chromosome alignment, leading to abnormal meiotic events (56, 59). However, interventions targeting mitochondrial function, such as phoenixin administration (61), MitoQ10 (68), and resveratrol supplementation (56), show promise in mitigating these effects. They regulate mitochondrial dynamics, enhance ovarian cell survival, and reduce apoptotic markers. Ultimately, they improve oocyte evolutionary and meiotic competence acquisition by lowering levels of ROS and lipid droplets, and by controlling mitochondrial distribution. Additionally, they contribute to reverse negative effects such as abnormal spindle morphology and chromosome arrangements in oocytes.

Notably, HFD is known to induce oxidative stress, which is implicated in DNA damage within oocytes. However, a study conducted by Shoaib et al. (66) provides promising evidence that a supplemental diet containing ferulic acid, kaempferol, malvidin, caffeoylquinic acid, and quercetin derivatives (*C. anthelminticum*) can effectively counteract these adverse effects. Specifically, this supplemental diet, when administered to rat model, has been shown to increase the activity of antioxidant enzymes such as catalase and superoxide dismutase (66). This, coupled with higher levels of glutathione and lower levels of malondialdehyde, plays a crucial role in protecting oocytes against DNA damage caused by reactive oxygen species (ROS), thereby reversing the harmful effects of the HFD (66). Similarly, interventions like MitoQ10 supplementation demonstrate efficacy in reducing cellular oxidative stress and improving mitochondrial function, thus mitigating DNA damage and preserving oocyte quality (68). Moreover, the influence of a HFD on the rigidity of the zona pellucida in oocytes within Graafian follicles is significant. Jia et al. have shown that targeted interventions can markedly enhance the rigidity of the zona pellucida. This improvement not only boosts oocyte meiosis but also mitigates oxidative stress, ultimately leading to the restoration of the oocytes' mechanical properties (56). These findings underscore the multifaceted impacts of HFD-induced oxidative stress on oocyte quality and highlight the importance of targeted interventions to ameliorate these effects, thereby preserving the oocyte quality and developmental potential.

#### 3.1.5 Biological matrices-derived antioxidants in HFD-induced oxidative stress: implications for reproductive lifespan

HFD-induced oxidative stress profoundly impacts reproductive lifespan, with diverse effects ranging from cyst formation to reproductive success. Cyst formation, a common consequence of HFD consumption, is mitigated by antioxidant interventions targeting oxidative stress which then demonstrate a decrease in the formation of follicular cysts, improving the reproductive potential (71, 72). Moreover, interventions aimed at reducing oxidative stress have shown promise in lowering the number of atretic follicles, inflammation, and apoptosis, thus ameliorating ovarian function and reproductive outcomes (56, 74).

Moreover, it is crucial to note that oxidative stress induced by a HFD has a significant impact on both the litter size and the survival rates of offspring. However, a study conducted by Wen et al. has shown that targeted interventions can effectively counteract these adverse effects. Specifically, the administration of phycocyanin into the mouse diet has been demonstrated to not only increase litter size but also improve offspring survival rates (70). This improvement is achieved by reducing follicular atresia and enhancing the levels of antioxidant enzymes in the ovaries, thereby mitigating the harmful effects of HFD-induced oxidative stress (70).

It is important to emphasize that the detrimental effects of obesity resulting from a HFD, can lead to infertility. However, in scenarios of obesity-induced infertility, improved reproductive success has been observed. This improvement has been achieved in a study conducted by Nilsson et al. (58) through interventions with organosulfur compounds such as α-lipoic acid, phytonutrients including forskolin and extracts of green coffee bean, green tea, and beetroot, vitamin E in the form of α-tocopheryl acetate, and Coenzyme Q10 (CoQ10) (58, 73). These interventions were applied in an obese rat model, specifically targeting oxidative stress, and led to significantly higher rates of reproductive success (58). These findings highlight the critical role of mitigating oxidative stress in enhancing fertility outcomes and ensuring the survival of offspring.

Furthermore, HFD-induced oxidative stress disrupts the ovarian cycle, impacting ovulation and subsequent successful fertilization and pregnancy rates. However, evidence in literature has shown that interventions targeting oxidative stress with ferulic acid, kaempferol, malvidin, caffeoylquinic acid, and quercetin derivatives (*C. anthelminticum*) (66) as well as the combined use of myo-inositol and α-lipoic acid (54), the diet administration of Gum Arabic (*Acacia senegal*) (65) and flavonoids like luteolin (69) hold promise in restoring normal ovarian cycle patterns (54, 66) and reduced ovarian degenerative changes (65) in rodent models, thus improving reproductive success in terms of fertilization and pregnancy rates (66, 69).

Overall, oxidative stress, induced by a HFD, has a profound impact on the reproductive lifespan, with inflammation and atresia being major concerns. Research has highlighted several antioxidant interventions that effectively mitigate these issues, thereby improving ovarian function and reproductive outcomes (56, 74). These interventions lead to a reduction in the number of atretic follicles, a decrease in ovarian inflammation, and a reduction in apoptosis. This is evidenced by decreased ovarian weights, reduced peri-ovarian fat pads, and modulated LH receptor positivity. Furthermore, the successful mitigation of inflammation and atresia is linked with improved reproductive success in obesity cases resulting from HFD. This underscores the critical importance of addressing oxidative stress in order to preserve ovarian health and fertility.

### 3.2 Nurturing ovarian resilience: perspectives in leveraging biological matrices as antioxidants amidst high-fat diets for female's reproductive wellbeing

#### 3.2.1 Clinical relevance: translating findings into female's health

The concept of nurturing ovarian resilience using biological matrices as antioxidants amidst HFD holds significant clinical relevance for female's reproductive health (75).

HFD have been associated with oxidative stress and inflammation, leading to impaired ovarian function and accelerated ovarian aging. Furthermore, oxidative stress and inflammation often interact synergistically, creating a vicious cycle that exacerbates ovarian dysfunction and compromises reproductive outcomes. ROS can stimulate inflammatory signaling pathways, while inflammatory mediators can induce the production of ROS, amplifying oxidative stress and inflammation in the ovaries. This crosstalk between oxidative stress and inflammation further contributes to the pathogenesis of ovarian disorders associated with HFD (4, 5). This results in reduced oocyte quantity and quality, negatively affecting fertility (29, 76, 77). Assisted reproductive technology (ART), developed in the late 20th century, has become a therapeutic solution to infertility issues (78). However, while ART can address the symptoms of fertility decline, it does not target the underlying cause related to HFD consumption.

This aspect is significant, as clinical studies have established a connection between the consumption of a HFD in women of reproductive age and the onset of conditions like endometriosis (79–83) and polycystic ovary syndrome (PCOS) (84–87). Both of these conditions can result in a diminished ovarian reserve and are among the primary contributors to infertility in women (88, 89). This identifies the urgent need to thoroughly study the ways in which HFD affects ovarian resilience and the overall reproductive system in order to identify the most suitable strategies to address it (78). Going into detail, endometriosis is a hormone-related, chronic inflammatory condition affecting women of childbearing age (90, 91). Despite ongoing research, the etiology and pathogenesis of endometriosis remain incompletely understood. Unlike some lifestyle diseases, the association between nutrition and the pathogenesis of endometriosis has been investigated and confirmed (90, 91). In a study conducted in 2018, Yamamoto et al. (79) found that prolonged consumption of saturated fats was associated with a 56% higher risk of developing endometriosis compared to those who are not accustomed to consuming them. Furthermore, Missmer et al. (80) demonstrated that women who regularly consumed trans fats were 48% more likely to develop endometriosis compared to those who did not. Although current research does not provide a clear dietary guideline or scientific recommendation for conditions related to endometriosis, it has been noted that omega-3 fatty acids, especially when combined with vitamin B12, can effectively alleviate symptoms related to endometriosis, particularly dysmenorrhea (81, 82). Furthermore, randomized controlled trials (RCTs) have demonstrated that *in vivo* administration of vitamins C and E resulted in reduced pelvic pain and lowered levels of inflammatory markers in peritoneal fluid compared to untreated patients. However, any beneficial effect of *in vivo* administration of vitamin C in patients with endometriosis did not positively impact on the *in vitro* fertilization (IVF) outcomes (83).

Regarding PCOS, it often presents with symptoms such as menstrual irregularities, absence of menstruation, and elevated androgen levels, along with associated metabolic dysfunctions (92–95). HFD have been linked to the development of metabolic disorders associated with PCOS and weight gain. This intensifies obesity and disrupts the hypothalamic-pituitary-ovarian axis through high adipokine production, causing impaired GnRH pulse due to overstimulation of kisspeptin neurons (5). At the pituitary level, there is abnormal release of gonadotropins, reflected locally in increased androgen production, which ovarian cells fail to fully convert into estrogen (92–95). This disruption triggers a cascade of events leading to increased insulin resistance, hyperinsulinemia, and the release of inflammatory adipokines. These factors contribute to an increase in fat synthesis and a decrease in fat breakdown, further exacerbating the metabolic and reproductive complications of PCOS (75, 92–95). Furthermore, PCOS patients typically exhibit heightened markers of oxidative stress in follicular fluid, reduced quality of oocytes and embryos (84), and elevated levels of chronic inflammation markers (85). While data on the efficacy of antioxidants to alleviate oxidative stress for enhancing fertility in PCOS patients remain limited (86), the administration of biological matrices combination comprising vitamin A, vitamin B1, vitamin B6, vitamin B12, vitamin C, vitamin D3, vitamin E, nicotinamide, and folic acid has shown promising results in improving pregnancy rates (87). Furthermore, two RCTs performed on patients with PCOS revealed that *in vivo* antioxidant treatment (specifically, resveratrol) improved the quality of oocytes and embryos (87). Additionally, treatments with vitamins D and E, respectively increased the rates of implantation and overall pregnancy success (96).

Oxidative stress induced by HFD seems to be linked with the development of hypertensive and metabolic disorders during pregnancy (97). A mild level of oxidative stress is a natural maternal response to pregnancy, due to the increased metabolic activity in the placental mitochondria, which is related to the growing fetus's higher metabolic demand (98). However, when the pregnancy-associated oxidative stress exceeds, it becomes detrimental to the pregnancy, creating conditions that can harm both the mother and the fetus (99). The pathological conditions caused by excessive oxidative stress during pregnancy encompass a range of hypertensive disorders, including hypertension in pregnancy, chronic hypertension, white-coat hypertension, masked hypertension, gestational hypertension, preeclampsia, proteinuria, and hemolysis with elevated liver enzymes and low platelet count (100). Furthermore, oxidative stress is implicated in the pathogenesis of gestational diabetes mellitus (101, 102). Clinical experience in using exogenous biological matrices as antioxidants for preventing and treating pregnancy complications is still in its early stages. One promising area of research is the potential use of melatonin in treating preeclampsia (103, 104). This interest is sparked by findings that lower melatonin levels are linked to the onset of this condition (105). In fact, studies have shown that administering melatonin to women suffering from severe early onset preeclampsia can significantly reduce oxidative damage to the maternal endothelium and extend the duration of pregnancy (106). Other antioxidants, such as vitamin C, vitamin E, and resveratrol, have been used in the management of preeclampsia. However, two large RCTs that tested vitamins C and E (107, 108) did not show any significant improvements. On the other hand, resveratrol showed promising results. It was found to be an effective supplement to oral nifedipine treatment for controlling blood pressure in women with preeclampsia (109, 110). Given these considerations, utilizing biological matrices as antioxidants represents a promising strategy to mitigate oxidative stress and enhance ovarian resilience. Translating these discoveries into clinical practice has the potential to revolutionize women's health. Strengthening ovarian resilience could extend women's reproductive lifespan, ultimately enhancing their overall wellbeing and quality of life. Furthermore, this approach could provide more control over reproductive health decisions, potentially leading to the development of personalized medicine strategies that consider individual variations in diet, lifestyle, and genetic factors.

In summary, the prospect of fortifying ovarian resilience using biological matrices as antioxidants in the context of HFD holds significant promise for advancing women's reproductive health. However, comprehensive research is essential to fully understand the underlying mechanisms and to effectively translate these findings into clinical interventions.

#### 3.2.2 Future directions: uncharted territories in understanding ovarian health

The concept of fostering ovarian resilience through the utilization of biological matrices as antioxidants, particularly in the context of a diet rich in fat, introduces new and unexplored avenues in the comprehensive understanding of ovarian health. As progress continues in this field, there are four key areas that warrant further investigation (111).

Firstly, the specific mechanisms by which an HFD induces oxidative stress and subsequently impacts ovarian function require thorough elucidation (76). This could entail a detailed examination of the role of specific dietary components, the influence of gut microbiota, and the intricate interaction between diet, inflammation, and hormonal regulation (112). Understanding these mechanisms will provide a foundation for developing targeted interventions to mitigate the adverse effects of an HFD on stepwise ovarian-mediated mechanisms (113, 114).

Secondly, while the potential of biological matrices as antioxidants is promising, additional research is necessary to identify the most effective matrices and comprehend their interaction with ovarian tissues (115). This could involve investigating a wide variety of biological matrices, ranging from vitamins (87) and minerals to plant extracts (116) and probiotics (117). The goal is to identify those matrices that not only have potent antioxidant properties but also interact synergistically with ovarian cells to enhance their resilience (118–120). In fact, the *in vitro* approach is indispensable for gathering data on potential impacts on the somatic and germinal compartments during development. This approach facilitates tracking of the effects induced on the quality of the egg cell throughout the initial stages of embryonic development. Notably, the use of 3D models in culture enables the extension of these observations to the development of embryos in the pre-implantation phase. Moreover, the employment of 3D culture models allows for the analysis of events occurring during the initial stages of ovarian folliculogenesis, and the tracking of their effects, from the gonadotropin-independent-sensitive phase to the gonadotropin-dependent phase.

Thirdly, before advancing to clinical trials, it is possible to collect a wealth of *in vivo* data from preclinical models. Ideally, these models should be developed on medium-sized monogastric mammals, as this closely mirrors the pathway of Non-Esterified Fatty Acids (NEFA) utilization in humans. The goal is not only to demonstrate the effectiveness of these interventions through rigorous clinical trials but also to understand how to seamlessly integrate them into existing treatment protocols and lifestyle recommendations (75). It is crucial to ensure that these interventions are not just effective, but also practical and well-received by patients (76, 118).

Finally, given the intricate relationship between diet, lifestyle, and ovarian health, a comprehensive approach is necessary. Future research should examine the influence of other lifestyle elements, such as physical activity and stress, on ovarian resilience, in connection with an unbalanced diet rich in fats (121). Moreover, the potential of personalized medicine strategies, considering individual variations in genetics, diet, and lifestyle, should be investigated (122). This approach acknowledges that each female is unique, and interventions must be customized to address their specific needs. In the light of this, the concept of nurturing ovarian resilience presents exciting new directions for understanding and enhancing female reproductive health. By further exploring these uncharted avenues, it is hoped that new strategies for promoting ovarian health and overall wellbeing can be discovered. These strategies will not only improve reproductive outcomes but also enhance the quality of life worldwide.

**Figure 3:**
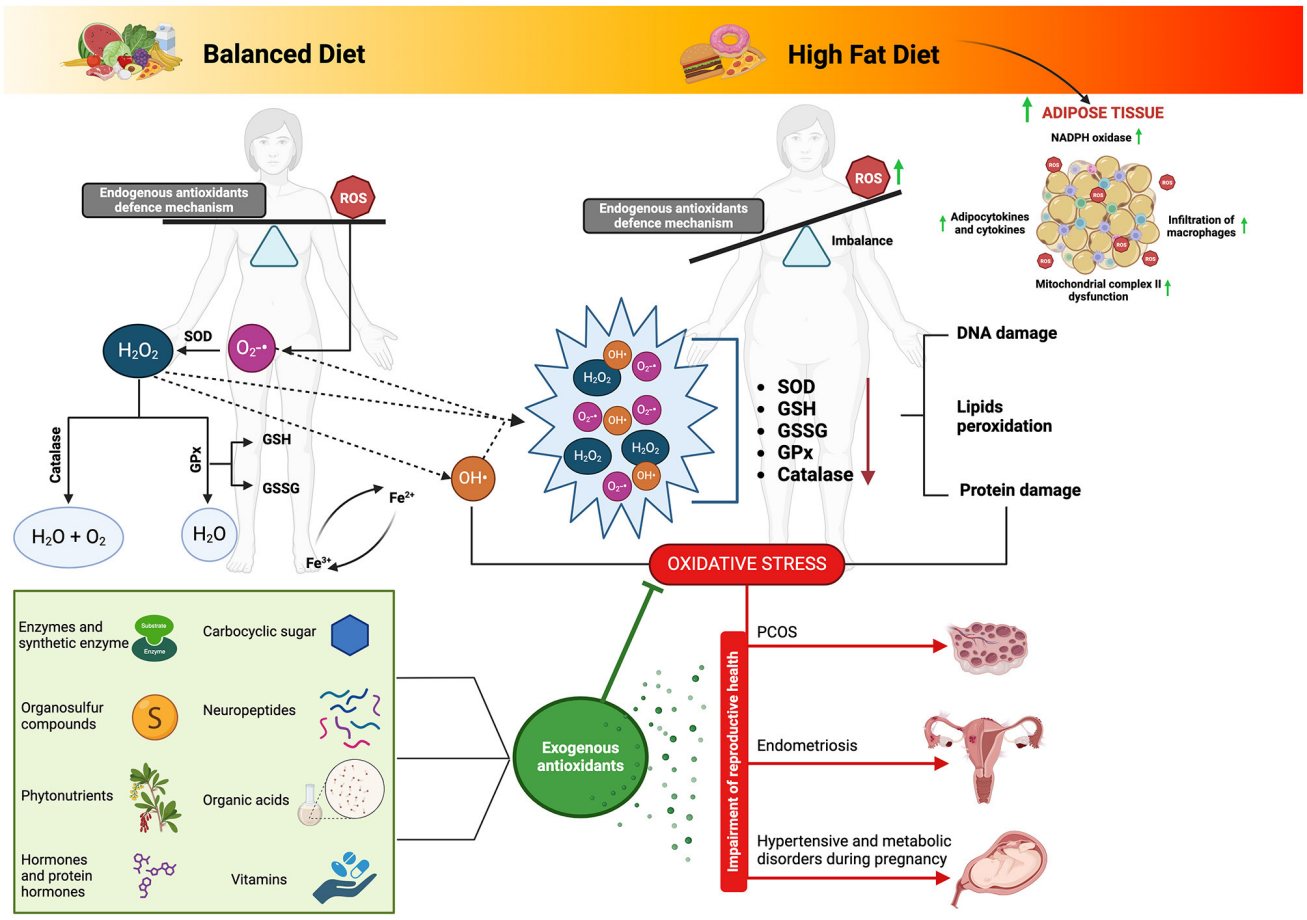
Biological matrices-derived antioxidants as dietary components: their influence on ovarian health and modulation of the complex interplay between high-fat diet-induced oxidative stress and reproductive function. Adipose tissue derived from a high-fat diet regimen can precipitate the production of reactive oxygen species (ROS) and subsequent oxidative stress through multifaceted pathways. Notably, the activation of nicotinamide adenine dinucleotide phosphate (NADPH) oxidase prompts ROS generation, fostering the upregulation of adipocytokines and cytokines, thus inciting inflammation and facilitating macrophage infiltration into adipose tissue. Furthermore, the heightened levels of ROS induce dysfunction within mitochondrial complex II, disrupting the electron transport chain and exacerbating ROS production. This intricate interplay underscores the pivotal role of oxidative stress in the pathophysiology of adipose tissue dysfunction amidst high-fat dietary habits. Connecting this phenomenon to fatty acid metabolism elucidates a complementary narrative, where electron transfer along the electron transport chain generates ROS as byproducts. Specifically, fatty acid metabolism within mitochondria serves as a prime site for ROS generation, with various complexes along the electron transport chain serving as key contributors. Consequently, the accrual of ROS can overwhelm endogenous antioxidant defenses, precipitating oxidative stress and consequent cellular damage. Such insights underscore the significance of biological matrices endowed with antioxidant properties, as they offer promising avenues for ameliorating the adverse effects of oxidative stress on physiological processes, including reproductive health. SOD, superoxide dismutase; GPx, glutathione peroxidase; GSSG, glutathione oxidase; GSH, glutathione reductase; ROS, reactive oxygen species; O_2_-•, superoxide; H_2_O_2_, hydrogen peroxide; OH•, Hydroxyl. Image created with Biorender.com.

A comprehensive overview of the topics discussed in this section can be found in Figure 3.

## 4 Discussion and concluding insights: the impact of antioxidants derived from biological matrices on ovarian health and reproductive wellbeing in the context of HFD

Oxidative stress, characterized by an imbalance where the production of ROS exceeds the body's antioxidant defenses, can significantly influence various health conditions. A diet high in saturated fats can trigger an overproduction of ROS, leading to oxidative stress, which may negatively impact female reproductive health. Different *in vivo* research studies on preclinical models have highlighted how the use of biological matrices with antioxidant properties can contribute to improving conditions associated with an HFD. This improvement is especially evident in the activation of steroidogenesis and the synchronized growth of both the somatic and germinal compartments of the ovarian follicle, resulting in enhanced oocyte quality.

However, further efforts are needed to understand the complex relationship between the potential of these biological matrices in promoting female reproductive health and mitigating the harmful effects of an HFD. These efforts require not only enhancing lab studies to examine the early stages of ovarian follicle development but also gathering extensive data from animal studies before advancing to human trials. With these insights, the concept of bolstering ovarian resilience presents exciting opportunities to deepen our understanding and enhance female reproductive health. As these yet-to-be-explored paths are investigated, it is expected that innovative approaches will emerge, improving overall reproductive wellbeing and enriching the quality of life world-wide.

## Data availability statement

All relevant data is contained within the article: The original contributions presented in the study are included in the article/Supplementary material, further inquiries can be directed to the corresponding author.

## Author contributions

CD: Data curation, Formal analysis, Investigation, Methodology, Writing – original draft, Writing – review & editing. UB: Data curation, Formal analysis, Investigation, Methodology, Writing – original draft, Writing – review & editing. CC: Data curation, Formal analysis, Investigation, Writing – review & editing. AP: Data curation, Formal analysis, Investigation, Writing – review & editing. GC: Data curation, Formal analysis, Investigation, Writing – review & editing. NB: Writing – review & editing. VR: Writing – review & editing. VG: Writing – review & editing. FK: Writing – review & editing. MD: Writing – review & editing. BB: Conceptualization, Data curation, Formal analysis, Funding acquisition, Investigation, Methodology, Project administration, Resources, Supervision, Validation, Visualization, Writing – original draft, Writing – review & editing.
