# Supplementary material for: High-fat diet-negative impact on female fertility: from mechanisms to protective actions of antioxidant matrices
Source: Front Nutr. 2024 Jun 10;11:1415455. doi: 10.3389/fnut.2024.1415455 (PMC11194403; doi:10.3389/fnut.2024.1415455)
Supplement: Supplementary file 1 [file Table_1.docx]

Supplementary Material

Supplementary Table 1: A summary of the existing literature review on antioxidant therapies targeting oxidative stress induced by high-fat diets and its impact on ovarian function in mammalian models. N/D: diet composition and effects not defined.

| Lipid diet composition | Dietary Supplements | | | Animal model | Experiment | Analysis of antioxidant action | Mechanism/site of action and biological effect | Effects on ovarian function | | | | References |
| --- | --- | --- | --- | --- | --- | --- | --- | --- | --- | --- | --- | --- |
|  | *Supplement* | *Bioactive compound category* | *Dose of supplement* |  |  |  |  | *Follicle Growth* | *Oocyte*  *quality* | *Steroidogenesis* | *Other effects* |  |
| N/D | myo-inositol | Carbocyclic sugar | 800 mg α-lipoic acid, 2 g myo-inositol/day | Human | *in vivo* | Total Antioxidant Capacity (TAC) in follicular fluid | Decreased levels of ROS and enhanced total antioxidant capacity in follicular fluid, leading to an improved oocyte environment. | N/D | The antioxidant status in follicular fluid is positively correlated with both successful fertilization and pregnancy rates. | N/D | N/D | (1) |
|  | α-lipoic acid | Organosulfur compound |  |  |  |  |  |  |  |  |  |  |
| Standard diet + 3% cholesterol, 8% cocoa butter, 2% cholic acid, 1% thiouracil and 1% starch. | Barley (*Hordeum vulgare*) and dates (*Phoenix dactylifera*) | Phytonutrients | Either 10% barley grains, 10% date palm fruit, or a combination of both (10% dates and 10% barley). | Rat | *in vivo* | Histopathological examination, immunohistochemical analysis of PCNA and caspase-3, assessment of CAT, SOD, GST, and MDA activities, and measurement of serum hormone levels. | Enhanced proliferation and maturation of healthy ovarian follicles, restoration of nearly normal cellular and structural patterns in the ovarian stroma, mitigation of oxidative stress and lipid peroxidation within the ovary, refinement, and amelioration of antioxidant levels in the assayed enzymes and sex steroid hormones. | Significant preservation of primordial, primary, preantral, and antral follicles, maintaining their numbers. | Significant protection of oocytes derived from mature follicles | Significant alterations and enhancements in the assayed hormone levels. | Significant decrease in the number of atretic follicles, with an additional anti-apoptotic effect observed in ovarian cells. | (2) |
| High-fat diet  (*diet composition N/D*) | Gum Arabic (*Acacia senegal*) | Phytonutrients  (fiber) | 10% weight/weight of gum arabic (GA) | Mouse | *in vivo* | Measurements included the assessment of antioxidant enzymes, malondialdehyde (MDA) levels, antioxidant enzyme activity, evaluation of ovarian histopathological changes, and quantification of mRNA expression of genes related to oxidative stress. | The intervention led to heightened activities of antioxidant enzymes such as superoxide dismutase, catalase, and glutathione peroxidase in the ovaries. Consequently, it mitigated oxidative stress and lipid peroxidation. Additionally, there was an increase in mRNA expression of antioxidant enzymes within the ovaries, which contributed to safeguarding against degenerative changes. | N/D | N/D | N/D | Degenerative changes in the ovary were diminished. | (3) |
| High-fat diet  (*diet composition N/D*) | Resveratrol | Phytonutrients  (phenol) | 10 mg/kg/day for 3 weeks | Mouse | *in vivo* | Analysis of oocyte quality encompassed assessments of metabolism, lipid accumulation, levels of ROS, progression of meiosis, alterations in spindle structure during Metaphase II, and mechanical measurement of zona pellucida (ZP) softness. | Enhanced oocyte meiosis, reinstated spindle assembly, and mitigated oxidative stress and irregular mitochondrial distributions within the oocyte. Furthermore, it restored the mechanical properties of oocytes, specifically the hardness of the zona pellucida. | N/D | Decrease in ROS levels, lipid droplet accumulation, and irregular distribution of mitochondria in the oocyte. Additionally, normalization of spindle morphology and chromosome arrangement within the oocyte. | N/D | Significant decrease in the number of atretic follicles and restoration of the softened zona pellucida of oocytes observed in obese mice. | (4) |
| The fat content of High-fat diet was adjusted to 60% by addition of beef tallow into standard diet | Okra (*Abelmoschus esculentus*) | Phytonutrients (flavonoid) | 200mg/kg for 30 days | Rat | *in vivo* | Assessment of serum antioxidant activity, including superoxide dismutase (SOD), glutathione peroxidase (Gpx), and serum catalase levels. Histological examination of ovarian tissue to study its structure and characteristics. Evaluation of ovarian expression levels of genes associated with apoptosis and anti-apoptosis. | The intervention led to a decrease in the numbers of atretic preantral and antral follicles in the ovary, accompanied by an increase in the activities of antioxidant enzymes such as glutathione peroxidase (GPX) and catalase (CAT). Furthermore, there was a reduction in lipid peroxidation levels and modulation of apoptotic gene expression in the ovaries. | There was a reduction in the number of preantral and antral atretic follicles, alongside an increase in the presence of new or active follicles. Additionally, normal follicular growth was restored. | It is possible that it plays a role in expediting follicular maturation by mitigating follicular atresia, potentially achieved through augmenting the ratio of antiapoptotic to apoptotic genes in the ovary. | N/D | There was a reduction in the number of atretic preantral and antral follicles. | (5) |
| High-fat diet comprising 40% fat, 20% protein, 36% carbohydrate, and 4% others (530 kcal) | Ferulic acid, kaempferol, malvidin, caffeoylquinic acid, and quercetin derivatives extracted from bitter cumin (*Centratherum anthelminticum*) using an ethanolic extraction method. | Phytonutrients (flavonoids and phenolic compounds) | 250, 500, or 750 mg/kg/day for 28 days | Rat | *in vivo* | Histological examination was conducted alongside measurements of sex hormone and inflammatory marker levels using ELISA. Additionally, catalase (CAT) and superoxide dismutase (SOD) activity, as well as malondialdehyde (MDA) and glutathione (GSH) levels, were assessed from tissue homogenate. | The intervention resulted in improved oxidative stress markers and catalase activity in the ovary, normalized estrous cycle, and balanced reproductive hormone levels. | The study revealed various follicles at different developmental stages, including growing follicles (GF), antral follicles (AF), and preantral follicles (PAF). Additionally, a corpus luteum was observed, accompanied by a decreased number of secondary follicles, indicating the restoration of fertility. | The increase in catalase (CAT) and superoxide dismutase (SOD) activity, coupled with higher levels of glutathione (GSH) and lower levels of malondialdehyde (MDA), collectively contribute to the protection of cells against reactive oxygen species (ROS), which have the potential to damage DNA. | There was an increase in follicle-stimulating hormone (FSH) and progesterone levels, accompanied by a decrease in luteinizing hormone (LH) levels. | The disappearance of cysts and the emergence of corpora lutea, indicative of improved ovulation, along with the presence of normal follicles, suggest an improvement in polycystic ovary syndrome (PCOS). | (6) |
| High-fat diet  (*diet composition N/D*) | Thymoquinone | Phytonutrients | TQ (10% pmm) and TQ (20% pmm) | Mouse | *in vivo* | Histopathological examination was conducted on mammary and ovarian samples, alongside evaluation of metabolic and oxidant status. Additionally, qRT-PCR analysis was performed to verify the activity of the AMPK/PGC1α/SIRT1 metabolic pathway. | The activated genes associated with the AMPK/PGC1α/SIRT1 pathway exerted a positive influence on oxidative status, leading to a reduction in inflammatory markers and enhancement of mitochondrial function in ovarian tissue. Additionally, they regulated oxidative stress biomarkers and antioxidant enzyme activities. | There was an increase in the number of Graafian follicles, ovulated oocytes, and corpus luteum formation. | N/D | N/D | N/D | (7) |
| High-fat diet containing 60% kcal energy prepared using lard. | Apple vinegar | Organic Acid | 5 g vinegar powder/100 g for 8 weeks | Rat | *in vivo* | Biochemical tests were conducted on serum samples, while sex hormones were determined using an ELISA kit. Ovary follicle counts were analyzed using histological methods, and the proliferation index of granulosa cells was determined through immunohistochemistry. Additionally, kisspeptin expression in the ovary was detected using RT-PCR. | The intervention led to an increase in the numbers of primordial and primary follicles in the ovary, as well as elevated serum levels of estradiol. There was a four-fold increase in serum total antioxidant capacity (TAC), and regulation of kisspeptin expression in the ovary was observed, along with its indirect effects on folliculogenesis. | There was an increase in the number of primordial and small primary follicles, as well as elevated ovarian kisspeptin expression, indirectly influencing folliculogenesis. | N/D | The intervention led to an increase in estradiol levels. | N/D | (8) |
| High-fat diet, provided 5.4 kcal per gram and consisted of 25.9% carbohydrates, 14.9% proteins, and 59.0% fat. | Phoenixin | Peptide (neuropeptide) | Administering 100 nmol/g body weight via gastrogavage for a duration of 10 weeks. | Rat | *in vivo* | Blood samples were collected for biochemical assessment of hormonal state, inflammation, oxidative stress (OS), and apoptosis. Mitochondrial samples were analyzed, and quantitative estimation of GnRH receptor (GnRHR) mRNA abundance was conducted. Additionally, relative abundance of mitochondrial-related proteins such as dynamin-related protein 1 (Drp1) and mitofusin 2 (Mfn2) genes were assessed using real-time PCR. Histopathological evaluation of ovarian tissues was also performed. | The intervention improved obesity-induced infertility by modulating mitochondrial dynamics, resulting in decreased serum levels of insulin and testosterone, as well as ovarian levels of certain proteins and markers associated with oxidative stress and apoptosis. Additionally, there was an increase in serum estrogen, progesterone, luteinizing hormone (LH), and follicle-stimulating hormone (FSH), along with elevated ovarian levels of GnRH receptor (GnRHR), mitofusin2 (Mfn2), mitochondrial transmembrane potential (ΔΨm), and electron transport chain (ETC) complex-I. | PNX-induced regulation of mitochondrial dynamics enhanced ovarian cell survival and decreased caspase-3 levels, indicating an anti-apoptotic effect. | N/D | The upregulation of GnRH receptor enhances ovarian steroidogenesis, resulting in decreased serum testosterone and insulin levels, and increased serum estrogen, progesterone, luteinizing hormone (LH), and follicle-stimulating hormone (FSH) levels. | The intervention modulates obesity-induced oxidative stress, while also upregulating the expression of GnRH receptor (GnRHR), thus promoting ovarian cell survival and reducing apoptosis. | (9) |
| High-fat/sucrose Western diet (HF; TD.190341) has a composition of 15.3% kcal from protein, 42.8% kcal from carbohydrates (with sucrose contributing 345 g/kg), and 41.9% kcal from fat. This diet provides 4.56 kcal per gram. | α-lipoic acid | organosulfur compound | 1 g/kg for 6 weeks | Mouse | *in vivo* | The study involved ovarian histopathology using hematoxylin and eosin staining, TUNEL staining to detect apoptotic cells, quantitative RT-PCR analysis to assess gene expression levels, observation of mating behavior, and evaluation of reproductive outcomes. | The improved reproductive success in obesity may be attributed, at least in part, to the mitigation of ovarian inflammation and the reduction of follicular atresia in the ovary. | N/D | N/D | N/D | Obesity-induced infertility was partially mitigated in the treated group, as evidenced by the presence of copulatory plugs, higher number of litters, and increased reproductive success compared to the control group. This improvement was associated with a reduction in ovarian inflammation and attenuated follicular atresia. | (10) |
|  | Green coffee bean extract | Phytonutrients | 2.5 g/kg for 6 weeks |  |  |  |  |  |  |  |  |  |
|  | Green tea extract | Phytonutrients | 0.75 g/kg for 6 weeks |  |  |  |  |  |  |  |  |  |
|  | Forskolin | Phytonutrients (labdane diterpenoid) | 0.125 g/kg for 6 weeks |  |  |  |  |  |  |  |  |  |
|  | Vitamin E/α tocopheryl acetate | Vitamin (Vitamin E) | 2.188 g/kg for 6 weeks |  |  |  |  |  |  |  |  |  |
|  | Beetroot extract | Phytonutrients (betalains) | 10 g/kg for 6 weeks |  |  |  |  |  |  |  |  |  |
|  | CoQ10 | Enzyme (ubiquinone) | 2.5 g/kg for 6 weeks |  |  |  |  |  |  |  |  |  |
| High-fat diet comprised 54.2% standard diet, 16.8% lard, 15% sucrose, 9% casein, 1% minerals, 1% vitamins, and 3% malt dextrin. | MitoQ10 | Synthetic enzyme | The treatment regimen involved administering 500 µmol/l of MitoQ10 (Sigma-Aldrich; Merck KGaA) daily for 8 consecutive weeks. | Rat | *in vivo* | Histopathology was employed to analyze the ovarian morphological changes. Endocrine and reproductive-related parameters were assessed using ELISA, western blotting, and measurement of oxidative stress-associated biomarkers. | The intervention reversed endocrine abnormalities, reduced oxidative stress, and improved mitochondrial function. It also led to a decrease in the expression of apoptotic proteins and improvement in reproductive and metabolic features. Additionally, there was a reduction in ROS levels. | N/D | N/D | The intervention resulted in decreased levels of testosterone, luteinizing hormone (LH), LH/FSH ratio, fasting insulin (FIN), and homeostatic model assessment of insulin resistance (HOMA-IR). | MitoQ10 has the potential to reduce cellular oxidative stress resulting from mitochondrial dysfunction, which is implicated in polycystic ovary syndrome (PCOS) insulin resistance. | (11) |
| High-fat diet  (*diet composition N/D*) | Luteolin | Phytonutrients (flavonoid) | The doses administered were 25, 50, and 100 mg/kg intraperitoneally daily. | Rat | *in vivo* | The study included serum sex hormone testing, western blot analysis, measurement of antioxidant activities, and Real-Time qPCR analysis. | The intervention normalized the estrus cycle, improved ovarian morphology, and balanced serum sexual hormone levels. It demonstrated inhibitory effects on insulin resistance by regulating the PI3K/AKT signaling pathway. Additionally, it restored the activities of antioxidants such as SOD, GPx, CAT, and GSH, and upregulated the Nrf2 pathway, contributing to an enhanced antioxidative response in the ovaries. | N/D | N/D | The intervention led to increased levels of serum follicle-stimulating hormone (FSH) and estradiol, while luteinizing hormone (LH) and testosterone levels decreased. | The intervention resulted in the normalization of the estrus cycle and improvement in ovarian morphology. This included a reduction in polycystic features and alleviation of the loss of oocytes and corpus luteum in PCOS rats. | (12) |
| High-fat diet (D12492, Research Diets Inc., New Brunswick, NJ) | Melatonin | Hormone (indoleamine) | The treatment regimen involved administering daily oral doses of 30 mg/kg body weight for 3 weeks. | Mouse | *in vivo/ in vitro* | The study utilized morpholino knockdown and acetylation-mimetic mutant overexpression assays, as well as in vitro fertilization (IVF) and embryo culture techniques. Additionally, ROS measurement, western blotting, and immunofluorescence were employed for analysis. | The intervention led to reduced reactive oxygen species (ROS) generation and prevented spindle/chromosome anomalies in oocytes. Consequently, it promoted the developmental potential of early embryos. During in vitro maturation, it also attenuated oxidative stress and meiotic defects in HFD oocytes through the SIRT3-SOD2-dependent mechanism, which is the site of action of melatonin. | N/D | The intervention reduces ROS generation and prevents spindle/chromosome anomalies in oocytes, thus promoting the developmental potential of early embryos. Additionally, during in vitro maturation, it markedly attenuates oxidative stress and meiotic defects in oocytes. | N/D | The intervention ameliorates defective phenotypes in oocytes induced by maternal obesity through the SIRT3-SOD2-dependent mechanism. | (13) |
| 240 g/d soya oil | Melatonin | Hormone (indoleamine) | The intake of 300 g/d of inulin and cellulose until the 19th day of the fourth estrous cycle led to an increase in serum levels of serotonin and melatonin, which were indirectly synthesized after dietary fiber intake. | Pig | *in vivo* | The study involved morphological classification of follicles, TUNEL assay for apoptosis detection, measurement of serum serotonin and melatonin levels, gene expression analysis, and western blotting for protein expression assessment. | The intervention protected against ovarian follicular atresia, at least partly through gut microbiota-related serotonin-melatonin synthesis. It resulted in decreased numbers of atretic follicles and lowered expression of apoptotic markers in the ovaries. | The intervention led to an increase in the number of primordial and antral follicles, resulting in a higher total number of follicles. | N/D | N/D | The intervention significantly decreased granulosa cell apoptosis and reduced the percentage of atretic antral follicles. | (14) |
|  | Seratonin | Hormone (monoamine neurotransmitter) |  |  |  |  |  |  |  |  |  |  |
| High-fat diet (D12492, Research Diets Inc., New Brunswick, NJ, United States) | Phycocyanin | Phytonutrients (biliprotein from *Spirulina platensis*) | 500 mg/kg/day | Mouse | *in vivo/ in vitro* | The study involved ovarian histology and follicle counting, RT-PCR and biochemistry assays, in vitro maturation (IVM), in vitro fertilization (IVF), and embryo culture techniques. Additionally, immunofluorescence was used for protein expression analysis, while mitochondria distribution and mitochondrial membrane potential were evaluated. Furthermore, reactive oxygen species (ROS) assay and annexin-V staining were conducted for apoptosis detection. | The intervention ameliorated the level of ovarian antioxidant enzymes, reduced the occurrence of follicular atresia, and improved both the abnormal morphology of the spindle-chromosome complex (SCC) and the abnormal mitochondrial distribution pattern in oocytes. Additionally, it partially reversed obesity-related accumulation of reactive oxygen species (ROS), decreased the number of early apoptotic cells, and normalized the expression of H3K9me3 in oocytes. Furthermore, it prevented ovarian follicular atresia, reduced follicle atresia rates, and improved fertility-related hormone levels. | N/D | The intervention led to the recovery of abnormal morphology of the spindle-chromosome complex and the abnormal mitochondrial distribution pattern in oocytes. Additionally, it partially reversed the accumulation of reactive oxygen species (ROS), reduced the number of early apoptotic cells, and normalized the abnormal expression of H3K9me3 in oocytes. | FSH levels decreased as a result of the intervention. | The intervention resulted in an increase in litter size and improved offspring survival rates. Additionally, it ameliorated the level of ovarian antioxidant enzymes and reduced follicular atresia in obese female mice. | (15) |
| The Metabolic Syndrome diet was prepared with additional refined palm oil (10%) and coconut oil (15%). | Betalain | Phytonutrients (betalains) | 300mg/kg for 2 months | Rat | *in vivo* | Performing histopathology and Immunohistochemistry to assess iNOS and Caspas-3 markers, along with biochemical analysis of plasma parameters associated with metabolic syndrome, will provide comprehensive insights into the underlying mechanisms. | There is a mild to moderate enhancement in ovarian function characterized by an augmented follicular count and reduced incidence of ovarian cyst formation. | There is a notable moderate enhancement observed in the quantity of ovarian follicles. | N/D | N/D | There is a noticeable reduction in the formation of follicular cysts. | (16) |
| High-fat diet formulated with supplementary refined palm oil (10%), coconut oil (15%), and cholesterol (1%) was prepared. | Frankincense (*Boswellia Carterii*) | Phytonutrients (oil extract) | 500 mg/kg body weight for 60 days | Rat | *in vivo* | Plasma samples were collected and subjected to analysis, while histopathological examination and immunohistochemistry were performed on genital organs. | The detrimental effects of a high-fat diet on reproductive organs were mitigated, evidenced by an increase in follicle count and a reduction in cyst formation. | There was a notable increase in the follicle count. | N/D | N/D | A decrease in the number of follicular cysts was observed, accompanied by a weak positive reaction of caspase-3 and iNOS in the granulosa cells of ovarian follicles and stromal cells. | (17) |
| Isocaloric high-fat/high-sugar (HF/HS) diet was administered using Test Diet 58R3 (TestDiet), comprising 59% fat, 17% sucrose, and 15% protein by weight. | CoQ10 | Enzyme (ubiquinone) | Administered three times per week, a dosage of 22 mg/kg dissolved in sesame oil was delivered subcutaneously for a duration of 6 weeks. | Mouse | *in vivo* | Oocytes were gathered and stained to evaluate mitochondrial distribution, quantify reactive oxygen species (ROS), assess meiotic spindle formation, and measure metabolites. Subsequent in vitro fertilization was conducted, and resulting blastocyst embryos were transferred into control mice. Parameters such as oocyte count, fertilization rate, blastulation rate, and implantation rate were then meticulously measured. | Mitochondrial distribution abnormalities were completely prevented, resulting in an increased percentage of normal spindle and chromosome alignment. Furthermore, there was notable improvement observed in both oocyte mitochondrial distribution and function. | N/D | It significantly enhances the mitochondrial function of oocytes, leading to a notable increase in the percentage of normal spindle and chromosome alignment. | N/D | N/D | (18) |
| High-fat diet comprises 40% of calories derived from fat, with a composition of 60% fat, 20% protein, and 20% carbohydrates. | Leptin | Protein hormone | N/D | Mouse | *in vivo* | Numerical values of follicles were estimated, and the levels of LH receptor (LHr) immune-positivity, catalase (CAT), and myeloperoxidase (MPO) were determined. | The observations included reduced ovarian weights, diminished peri-ovarian fat pads, modulation of LH receptor positivity, decreased apoptosis and inflammation, along with increased LH receptor positivity in the ovary. | In the leptin groups, the volume of the granulosa layer was significantly higher. Additionally, in all treated groups, the volume of the theca layer and the volume of the antrum were significantly increased. There was also observed an increase in LHR immunoreactivity in both granulosa and theca cells, which accelerated follicular turnover and increased the number of dominant follicles. | N/D | There was an observed increase in the mean LH concentration. | The treatment resulted in a reduction of apoptosis and inflammation, evidenced by a lower number of atretic follicles in the treated groups. | (19) |

**References**

1. Novielli C, Anelli GM, Lisso F, Marzorati A, Parrilla B, Oneta M, Savasi VM, Cetin I, Mandò C. Effects of α-lipoic acid and myo-inositol supplementation on the oocyte environment of infertile obese women: A preliminary study. *Reprod Biol* (2020) **20**:541–546. doi:10.1016/J.REPBIO.2020.10.002

2. El-Sayyad HIH, El-Shershaby EMF, El-Mansi AA, El-Ashry NE. Anti-hypercholesterolemic impacts of barley and date palm fruits on the ovary of Wistar albino rats and their offspring. *Reprod Biol* (2018) **18**:236–251. doi:10.1016/J.REPBIO.2018.07.003

3. Ahmed AA, Fedail JS, Musa HH, Musa TH, Sifaldin AZ. Gum Arabic supplementation improved antioxidant status and alters expression of oxidative stress gene in ovary of mice fed high fat diet. *Middle East Fertil Soc J* (2016) **21**:101–108. doi:10.1016/J.MEFS.2015.10.001

4. Jia Z, Feng Z, Wang L, Li H, Wang H, Xu D, Zhao X, Feng D, Feng X. Resveratrol reverses the adverse effects of a diet-induced obese murine model on oocyte quality and zona pellucida softening. *Food Funct* (2018) **9**:2623–2633. doi:10.1039/C8FO00149A

5. Majd NE, Azizian H, Tabandeh M, Shahriari A. Effect of Abelmoschus esculentus Powder on Ovarian Histology, Expression of Apoptotic Genes and Oxidative Stress in Diabetic Rats Fed with High Fat Diet. *Iran J Pharm Res* (2019) doi:10.22037/IJPR.2019.2325

6. Shoaib M, Saleem A, Zeb A, Khan MI, Akhtar MF. Chemical Characterization and Ameliorating Effect of Centratherum anthelminticum Extract against Polycystic Ovary Syndrome in Wistar Rats. *Int J Endocrinol* (2023) **2023**: doi:10.1155/2023/4978562

7. Harphoush S, Wu G, Qiuli G, Zaitoun M, Ghanem M, Shi Y, Le G. Thymoquinone ameliorates obesity-induced metabolic dysfunction, improves reproductive efficiency exhibiting a dose-organ relationship. *Syst Biol Reprod Med* (2019) **65**:367–382. doi:10.1080/19396368.2019.1626933

8. Shams F, Aghajani-nasab M, Ramezanpour M, Fatideh RH, Mohammadghasemi F. Effect of apple vinegar on folliculogenesis and ovarian kisspeptin in a high-fat diet-induced nonalcoholic fatty liver disease in rat. *BMC Endocr Disord* (2022) **22**:1–13. doi:10.1186/S12902-022-01205-1/FIGURES/6

9. Basha EH, Eltokhy AKB, Eltantawy AF, Heabah NAE, Elshwaikh SL, El-Harty YM. Linking mitochondrial dynamics and fertility: promoting fertility by phoenixin through modulation of ovarian expression of GnRH receptor and mitochondrial dynamics proteins DRP-1 and Mfn-2. *Pflugers Arch Eur J Physiol* (2022) **474**:1107–1119. doi:10.1007/S00424-022-02739-Y/FIGURES/8

10. Nilsson MI, May L, Roik LJ, Fuda MR, Luo A, Hettinga BP, Bujak AL, Tarnopolsky MA. A Multi-Ingredient Supplement Protects against Obesity and Infertility in Western Diet-Fed Mice. *Nutrients* (2023) **15**:611. doi:10.3390/NU15030611/S1

11. Ding Y, Jiang Z, Xia B, Zhang L, Zhang C, Leng J. Mitochondria-targeted antioxidant therapy for an animal model of PCOS-IR. *Int J Mol Med* (2019) **43**:316–324. doi:10.3892/IJMM.2018.3977

12. Huang Y, Zhang X. Luteolin alleviates polycystic ovary syndrome in rats by resolving insulin resistance and oxidative stress. *Am J Physiol Endocrinol Metab* (2021) **320**:E1085–E1092. doi:10.1152/AJPENDO.00034.2021

13. Han L, Wang H, Li L, Li X, Ge J, Reiter RJ, Wang Q. Melatonin protects against maternal obesity-associated oxidative stress and meiotic defects in oocytes via the SIRT3-SOD2-dependent pathway. *J Pineal Res* (2017) **63**: doi:10.1111/JPI.12431

14. Gong Y, Tang L, Jiang X, Li Y, Yang M, Xu S, Li J, Che L, Lin Y, Feng B, et al. Gut microbial metabolism of dietary fibre protects against high energy feeding induced ovarian follicular atresia in a pig model. *Br J Nutr* (2021) **125**:38–49. doi:10.1017/S0007114520002378

15. Wen X, Han Z, Liu SJ, Hao X, Zhang XJ, Wang XY, Zhou CJ, Ma YZ, Liang CG. Phycocyanin Improves Reproductive Ability in Obese Female Mice by Restoring Ovary and Oocyte Quality. *Front cell Dev Biol* (2020) **8**: doi:10.3389/FCELL.2020.595373

16. Alshafei MM, Mohammed DM, Hanafi EM, Kassem SS, El-Messery TM, Korany RMS, El-Said MM. Red Beet Peel-Loaded Liposome’s Impact on Metabolic Syndrome Rats and Its Use as Fortifier in Yoghurt Powder. *Egypt J Chem* (2023) **66**:517–533. doi:10.21608/EJCHEM.2022.129208.5714

17. Hanafi EM, Korany RMS, Tawfeek FM, Hozyen HF, El Natat WS, Ramadan MM, Kasem SS, Alshafei MM, Mohammed DM. Effect of Frankincense (Boswellia Carterii) on Animal Reproductive Performance. *Egypt J Chem* (2023) **66**:213–223. doi:10.21608/EJCHEM.2022.138276.6093

18. Boots CE, Boudoures A, Zhang W, Drury A, Moley KH. Obesity-induced oocyte mitochondrial defects are partially prevented and rescued by supplementation with co-enzyme Q10 in a mouse model. *Hum Reprod* (2016) **31**:2090–2097. doi:10.1093/HUMREP/DEW181

19. Tümentemur G, Altunkaynak BZ, Kaplan S. Is melatonin, leptin or their combination more effective on oxidative stress and folliculogenesis in the obese rats? *J Obstet Gynaecol (Lahore)* (2020) **40**:116–127. doi:10.1080/01443615.2019.1657816
