# Supplementary figures and images for: High-fat diet-negative impact on female fertility: from mechanisms to protective actions of antioxidant matrices
Source: Front Nutr. 2024 Jun 10;11:1415455. doi: 10.3389/fnut.2024.1415455 (PMC11194403; doi:10.3389/fnut.2024.1415455)

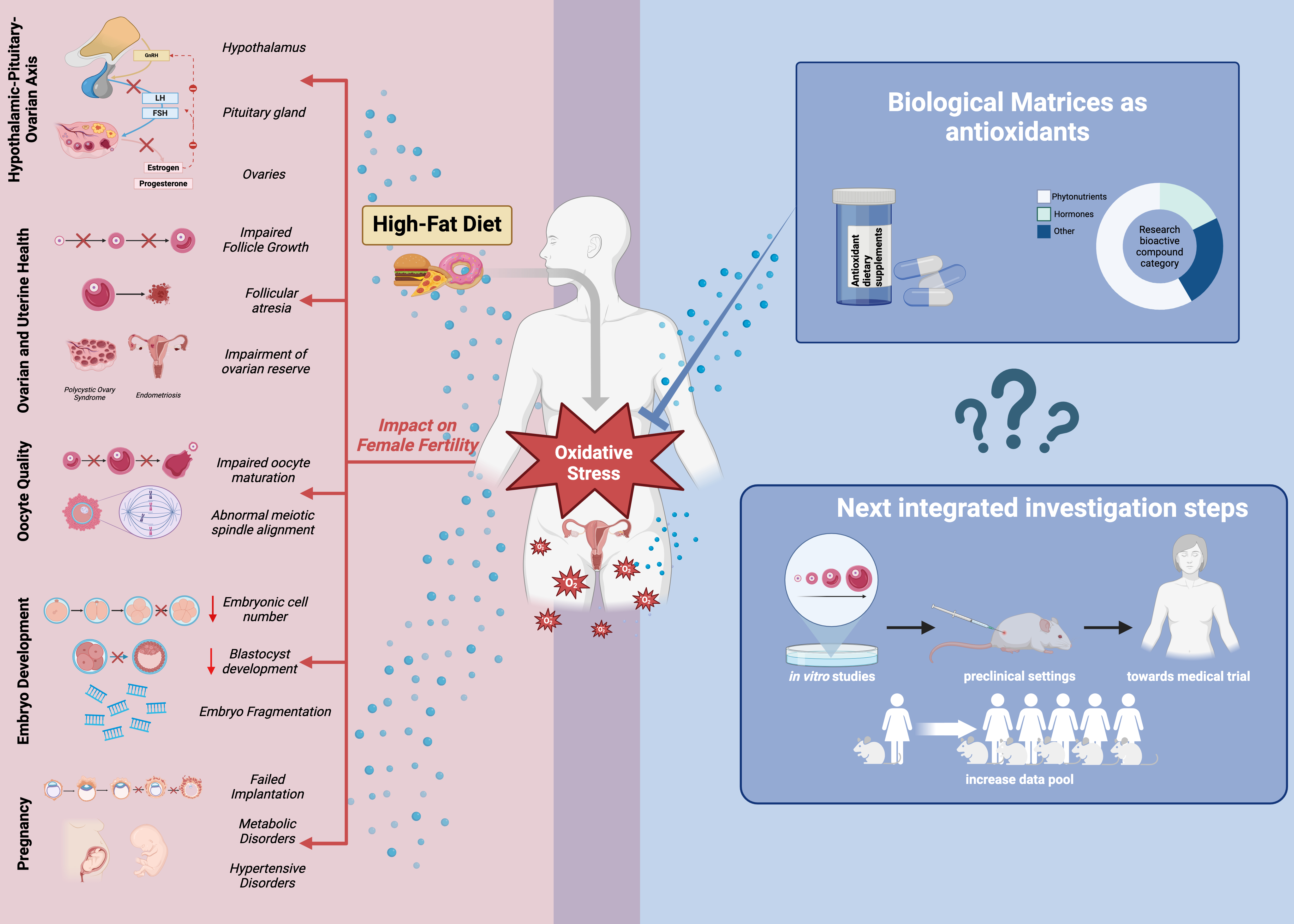

Supplement: Supplementary file 2 [file Image_1.JPEG]
